# iPAR: a new reporter for eukaryotic cytoplasmic protein aggregation

**DOI:** 10.1186/s44330-025-00023-w

**Published:** 2025-04-01

**Authors:** Sarah Lecinski, Jamieson A. L. Howard, Chris MacDonald, Mark C. Leake

**Affiliations:** 1 School of Physics, Engineering and Technology, University of York, York, YO10 5DD UK; 2 Department of Biology, University of York, York, YO10 5DD UK; 3 York Biomedical Research Institute, University of York, York, YO10 5DD UK

**Keywords:** *Saccharomyces cerevisiae*, Protein aggregation, Inheritance, Cell ageing, Confocal microscopy, Single-molecule

## Abstract

**Background:**

Cells employ myriad regulatory mechanisms to maintain protein homeostasis, termed proteostasis, to ensure correct cellular function. Dysregulation of proteostasis, which is often induced by physiological stress and ageing, often results in protein aggregation in cells. These aggregated structures can perturb normal physiological function, compromising cell integrity and viability, a prime example being early onset of several neurodegenerative diseases. Understanding aggregate dynamics *in vivo* is therefore of strong interest for biomedicine and pharmacology. However, factors involved in formation, distribution and clearance of intracellular aggregates are not fully understood

**Methods:**

Here, we report an improved methodology for production of fluorescent aggregates in model budding yeast which can be detected, tracked and quantified using fluorescence microscopy in live cells. This new openly-available technology, iPAR (inducible Protein Aggregation Reporter), involves monomeric fluorescent protein reporters fused to a ∆ssCPY* aggregation biomarker, with expression controlled under the copper-regulated *CUP1* promoter

**Results:**

Monomeric tags overcome challenges associated with non-physiological reporter aggregation, whilst *CUP1* provides more precise control of protein production. We show that iPAR and the associated bioimaging methodology enables quantitative study of cytoplasmic aggregate kinetics and inheritance features *in vivo*. We demonstrate that iPAR can be used with traditional epifluorescence and confocal microscopy as well as single-molecule precise Slimfield millisecond microscopy. Our results indicate that cytoplasmic aggregates are mobile and contain a broad range of number of iPAR molecules, from tens to several hundred per aggregate, whose mean value increases with extracellular hyperosmotic stress

**Discussion:**

Time lapse imaging shows that although larger iPAR aggregates associate with nuclear and vacuolar compartments, we show directly, for the first time, that these proteotoxic accumulations are not inherited by daughter cells, unlike nuclei and vacuoles. If suitably adapted, iPAR offers new potential for studying diseases relating to protein oligomerization processes in other model cellular systems.

**Supplementary Information:**

The online version contains supplementary material available at 10.1186/s44330-025-00023-w.

## Introduction

Accumulation of misfolded protein aggregates is triggered by environmental stress conditions, which in turn compromise cell function. However, cells have evolved to respond to these changes to maintain metabolic function and ensure survival. In eukaryotic cells, systems such as the temporal protein quality control (PQC) sustain the proteome and actively contribute to the detection of misfolded proteins [1, 2], promoting their refolding mediated by chaperone proteins [2, 3]. The degradation of damaged proteins is actively mediated by the ubiquitin-proteasome system (UPS) [4, 5] but not all proteins are recognised this way, and other selective processes exist to degrade proteins, such as the autophagy pathway [6]. Generally, these systems require acute control of the temporal and spatial dynamics of subcellular components for quality control *in vivo* to prevent or clear aggregates and maintain proteomic homeostasis [2, 3, 5].

When quality control responses and processes fail, misfolded proteins accumulate in the intracellular environment with a heterogeneous size distribution of aggregates [7, 8], consistent with diffusion-nucleation mechanisms of formation [9]. This distribution of protein aggregates is harmful to the cell [10, 11], with endogenous protein aggregation effectively depleted from the cellular environment. Further toxicity is mediated by aggregation through perturbation of other functional proteins present in the crowded intracellular environment [12, 13]. Ultimately, this can lead to pathogenic phenotypes [14, 15]. Many neurodegenerative diseases (e.g. Parkinson's and Alzheimer's) are associated with a process which involves aggregation of amyloid resulting in packed beta-sheet structures and fibres [16–18], due in part to amyloid-β oligomerization [19]. Other diseases such as cataracts [20] and Huntington’s disease [21] result from the formation of amorphous aggregates [22, 23]. Understanding the formation of such proteotoxic factors is crucial to elucidating underlying mechanisms associated with cellular malfunction and toxicity. Insight into the associated *in vivo* dynamics of these factors can also contribute to the development of new therapeutic methods.

Budding yeast, *Saccharomyces cerevisiae*, has been used to investigate several important processes affecting intracellular organisation which are highly conserved across all eukaryotes, including key survival mechanisms [24, 25], essential metabolic pathways such as DNA replication [26, 27], transcription [28, 29], membrane trafficking [30–33], and PQC machinery for aggregate detection and clearance [3, 34, 35]. Considering its excellent genetic tractability, and ease of cell culturing and optical imaging, we used *S. cerevisiae* as a eukaryotic cellular model to investigate intracellular dynamics of aggregation. Various markers for aggregation use key conserved proteins present in yeast. Chaperone proteins are a good example of this; considered a first response against misfolded proteins, they are recruited at the site of misfolded proteins or aggregates to promote re-folding or initiate degradation pathways if necessary [36, 37]. Current approaches to analysing and quantifying protein aggregates include optical microscopy with use of fluorescent biomarkers of aggregation, typically using chaperone proteins as reporters (e.g. Hsp70, Hsp40, Hsp104) [20, 38–40]. Additionally, variants prone to form aggregates have been fluorescently tagged, such as the thermosensitive mutant of Ubc9 [41] derived from a SUMO-conjugating enzyme and unable to properly fold in yeast cells [42].

**Fig 1:**
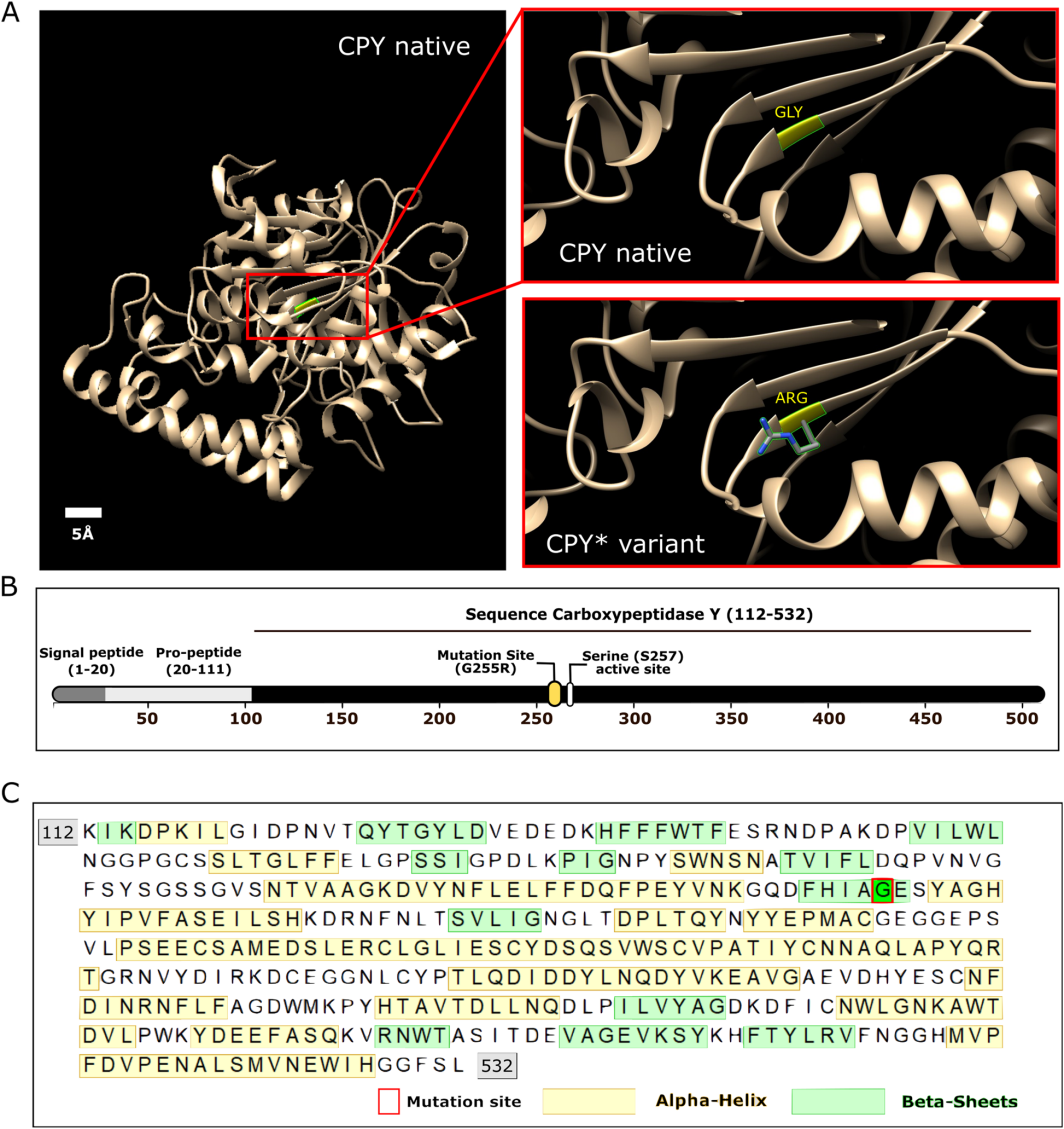
Modifications of CPY to enable its use as a reporter of cytoplasmic protein aggregation. **A** Left; a 3D model of the native CPY structure. Right; zoom-in of the mutated region, showing Glycine residue 255 in the native protein and the arginine substitution in the misfolded CPY* variant. Both amino acids are indicated in yellow. The 3D crystal structure of CPY (PDB ID: 1WPX) was visualised using Chimera software. **B** CPY sequence showing G255R mutation site near the S257 active site, responsible for the protein unfolding and aggregative behaviour. **C** Sequence for CPY, mutation site and native secondary structures. A red rectangle indicates the position of the mutation site (G255), alpha-helix regions in the native protein are shown in yellow and beta-sheets regions are displayed in green

Another common marker for aggregation used in *S. cerevisiae* is the engineered reporter ∆ssCPY*, a misfolded version of the vacuolar enzyme carboxypeptidase Y (CPY), which is prone to form aggregates and mis-localises to the cytoplasm [43, 44]. This variant, derived from the native CPY [45, 46], carries a single amino acid mutation with a glycine to arginine substitution at residue position 255 (G255R) [44, 47] (Figure 1). This mutation (labelled CPY*) is responsible for its misfolding, and when combined with an N-terminal truncated signal peptide (∆ss) results in aberrant localisation of this misfolded protein to the cytoplasm. Tagging of ∆ssCPY* with enhanced GFP (EGFP) has been used as a model to uncover PQC [48–50] and protein sorting dynamics [40, 51], cellular perturbations and protein aggregation kinetics in stressed cells [52, 53]. Studies have revealed that protein aggregate interactions and localisation *in vivo* have a crucial role in establishing toxicity [53].

The ∆ssCPY* aggregation reporter is typically expressed from the endogenous *PRC1* promotor, which is problematic as this gene is metabolically regulated, for example being upregulated under certain stress conditions, such as nutrient starvation [54, 55]. As protein aggregation correlates with cellular abundance of proteins and local protein concentrations, and is often assessed under stress conditions, there are challenges in disentangling phenotypes which are associated with metabolic-dependent expression and protein aggregation in such experiments. Furthermore, EGFP, and indeed several other fluorescent protein tags, has the capacity to dimerize [56–58], which can also potentially introduce challenging artifacts when assessing the aggregation of tagged molecules.

To address the limitations of existing aggregation biomarkers, we present newly developed versions of ∆ssCPY* as reporters for cytoplasmic protein aggregation that are tagged with monomeric fluorescent proteins and are expressed under the control of an inducible promoter. This new class of novel reagent, which we denote as an inducible Protein Aggregation Reporter (iPAR) is part of a useful methodology when used in conjunction with a range of fluorescence microscopy modalities to study several mechanistic aspects of stress-induced protein aggregation in cells.

For iPAR, we replaced the metabolically regulated endogenous promoter (*PRC1*) used to express ∆ssCPY* as reporter with the copper inducible promoter (*CUP1)*. The fluorescent fusion tag EGFP was additionally mutated to a monomeric version (mEGFP), which uses electrostatic repulsion to inhibit interactions between pairs of fluorescent protein molecules thereby minimising tag-induced oligomerisation effects. To enhance the utility of this new aggregation reporter with newer developed fluorescent proteins that have brighter fluorescence signal properties and faster maturation times than EGFP [59], we also constructed two variants of iPAR by swapping the mEGFP fusion tag with a brighter green monomeric fluorescent protein mNeonGreen as well as with the red fluorescent protein mScarlet-I. To increase the wider utility of this methodology for researchers, we further made these probes available for expression in budding yeast by creating plasmids of all three iPARs with both *URA3* and *LEU2* selection markers. Using these reagents with newly designed image analysis techniques, we were able to quantify the induced protein aggregation following hyperosmotic and elevated temperature cell stresses, and also to assess the capacity for mother cells to retain protein aggregates during the process of asymmetric cell division, during which other cellular organelles such as the nucleus and lytic vacuole are inherited in budding daughter cells. We further visualise iPAR *in vivo* using Slimfield microscopy, a rapid fluorescence imaging modality which can detect single fluorescent dye molecules including fluorescent proteins in the cytoplasm of a range of organisms including single bacteria [9, 60–67], yeast [68–70], algae [71, 72] and mammalian [73, 74] cells, as well as animal [75] and plant [76] tissue, with below millisecond sampling capability [77]. Analysis of iPAR aggregate Slimfield tracks indicate that aggregates are mobile in the vacuolar and nuclear compartments and possess between a few tens and a few hundred iPAR molecules per aggregate whose mean value increases upon extracellular hyperosmotic stress.

Here we describe the design and construction of iPAR and the associated molecular cloning and bioimaging methodology and demonstrate the method’s utility to improvement the reliability of cytoplasmic protein aggregation investigations. We make iPAR openly accessible as a resource to the research community.

## Material and methods

### Strains and plasmids used in the study

The yeast cell strains and plasmids used in this study are listed in Tables 1 and 2, respectively and the oligonucleotides used in Table 3.

**Table 1:** List of background yeast strains used in this study

**Parental strain**	**Genotype**	**Reference**	**Figure used**
BY4742	MAT**α**, *his3∆ leu2∆ lys2∆ ura3∆*	Brachmann, et al. 1998 [78]	Figs. 2- 5
BY4741	MAT**A**, *his3∆ leu2∆ met15∆ ura3∆*	Brachmann, et al. 1998 [78]	Fig. 5
BY4741	MAT**A**, *his3∆ leu2∆ met15∆ ura3∆* *NRD1*-mCherry::hgrB	Shashkova, et al. 2021 [69]	Fig. 5
BY4741	MATa *his3Δ1 leu2Δ0 met15Δ0 ura3Δ0* sfGFP-Hof1::*URA3*	(Weill et al., 2018) [79]	Supplementary Fig.4

**Table 2:** List of plasmids used in this study

**Plasmid**	**Genotype**	**Reference**	**Figure Used**
pLS190	pRS316 expressing ΔssCPY*-EGFP from the *PRC1* promoter	Stolz and Wolf, 2012 [44]	Fig. 2
pCM695	pRS316 expressing -GFP from the *CUP1* promoter	Laidlaw et al., 2021 [80]	Fig. 2
pLS191	pRS316 expressing ΔssCPY*-mEGFP from the *CUP1* promoter	This study	Figs. 2- 5
pLS195	pRS316 expressing ΔssCPY*-mScarlet-I from the *CUP1* promoter	This study	Fig. 4
pLS196	pRS316 expressing ΔssCPY*-mNeonGreen from the *CUP1* promoter	This study	Fig. 4
pCM264	Mup1-EGFP from the *MUP1* promoter	MacDonald *et al*., 2015 [81]	Supplementary Fig. 1
pLS199	pRS315 expressing ΔssCPY*-mEGFP from the *CUP1* promoter	This study	Not applicable
pLS200	pRS315 expressing ΔssCPY*-mScarlet-I from the *CUP1* promoter	This study	Not applicable
pLS198	pRS315 expressing ΔssCPY*-mNeonGreen from the *CUP1* promoter	This study	Not applicable

**Table 3:** Primers used for the construction of the initial iPAR fusion construct *CUP1*-ΔssCPY*-mEGFP and subsequent variants using mScarlet-I and mNeonGreen fluorescent proteins

**Oligo Name**	**Sequence (5’-3’)**	**Description**
cm193	GATATTAAGAAAAACAAACTGTAACGAATTCATGATCTCATTGCAAAGACCG	*CUP1*- ΔssCPY* - Forward primer - used to synthesise ΔssCPY* sequence for Gibson Assembly in pCM695
cm194	AGAATCGAGTTAAAAGGTATTGATTTTAAAGAAGATGGAAACGTTCTTGGACAC	ΔssCPY*-EGFP - reverse primer - used to synthesise ΔssCPY* sequence for Gibson Assembly in pCM695
S1	CACACAATCTAAACTTTCGAAAGATCC	EGFP - Forward primer - used to induce site directed mutagenesis (EGFP to mEGFP)
S2	CAGACAACCATTACCTGTC	EGFP – Reverse primer – used to induce site directed mutagenesis (EGFP to mEGFP)
S8	CCACGGTGGTTTCTCCTTACTCGAGAGTAAAGGAGAAGAACTTTTCACTGG	Forward primer - *Xho*I site Gibson assembly for mEGFP
S9	CCAGATATTCTATGGCAAAGCTTTTATTTGTATAGTTCATCCATGCC	Reverse primer- *Hind*III site Gibson assembly for mEGFP
S5	GGTGTTTCCAACACTGTCGCCGCTGGTAAGG	ORF ΔssCPY* - Forward sequencing primer
S25	AACTAATTACATGATATCGACAAAGGAAAA	Reverse sequencing primer in the CPY terminator - for verification of the EGFP sequence
S3	GGCAGACAAACAAAAGAATGG	Forward sequencing primer in mEGFP sequence - used to verify mEGFP site-directed mutagenesis.
cm3	TGTATCAATTGCATTATAATATCTTCTTGT	Forward sequencing primer in the *CUP1* promoter - used to verify the ΔssCPY* sequence
S14	GGTGGTTTCTCCTTACTCGAGATGGTGAGCAAGGG	Forward primer - *Xho*I site Gibson assembly for mScarlet-I
S15	CCAGATATTCTATGGCAAAGCTTCTACTTGTACAGCTCGTCC	Reverse primer - *Hind*III site Gibson assembly for mScarlet-I
S16	CCACGGTGGTTTCTCCTTACTCGAGATGGTCTCCAAAGGAGAGGCC	Forward primer - *Xho*I site Gibson assembly for mNeonGreen
S17	CCAGATATTCTATGGCAAAGCTTTTATTTATACAGCTCATCC	Reverse primer - *Hind*III site Gibson assembly for mNeonGreen

### Plasmid construction

An initial iPAR fusion construct *CUP1*-ΔssCPY*-mEGFP was generated using several cloning steps. Initially, the parent plasmid encoding *PRC1*-ΔssCPY*-EGFP (pLS190) was modified by site-directed mutagenesis using the S1 and S2 primers to incorporate the monomeric A206K mutation in EGFP [82]. This template was then used to amplify ΔssCPY* (oligos cm193 and cm194) and mEGFP (oligos S8 and S9) with compatible regions for Gibson Assembly [83]. ΔssCPY*-mEGFP was recombined between the *CUP1*-promoter and the *CYC1* terminator of pCM690 linearized with *Eco*RI and *Hind*III to generate pLS191 (C*UP1*-ΔssCPY*-mEGFP). The assembly strategy introduced 5’ *Xho*I and 3’ *Hind*III restriction sites flanking mEGFP. Plasmid pLS190 was linearized with *Xho*I and *Hind*III and Gibson assembly was used to exchange mEGFP with mScarlet-I [84] (using oligos S14 and S15, and using two PCRs separately to generate the *Xh*oI site) and mNeonGreen [85] (using oligos S16 and S17) variants of iPAR (respectively denoted as plasmids pLS195 and pLS196).

To maximise downstream applications of iPAR, in addition to creating red and green fluorescent variants with brighter fast maturing fluorescent proteins, we also switched the auxotrophic marker genes for plasmid selection (from *URA3* to *LEU2* selection). This was achieved by generating the *LEU2* gene from the integration plasmid pRS305, including ~300bp of plasmid common to the ΔssCPY* reporter expression plasmid to facilitate recombination (based on pRS316). The PCR product was transformed into wild-type yeast alongside the *URA3* expression plasmid allowing the marker to be converted to *LEU2* by homologous recombination (see Supplementary Information).

### Site-directed mutagenesis

The NEB Q5® Site-Directed Mutagenesis Kit (part number: E0554S, New England Biolabs Inc.) was used to perform the mutation responsible for mEGFP following the manufacturer’s protocol, with designed primers (S1 and S2, see Table 3) used at a concentration of 10 µM and the template DNA at a concentration between 1 to 25 ng/µl. The reaction mix was incubated for 5 min at room temperature before bacterial transformation.

### Gel DNA extraction

To extract linearized plasmid backbones, gel DNA extraction was performed using the “QIAquick Gel extraction kit” (part number: 28706X4, QIAGEN, Ltd.), following the supplier’s instructions. In short, the DNA band of interest (cut from the agarose gel following electrophoresis) was transferred into a sterile 1.5 ml Eppendorf tube. QG buffer was added to the tube to dissolve the gel (at a 3:1 volume proportion) and incubated for 10 min at 50°C. The sample was loaded onto a silica-membrane-based spin column (1.5 ml volume) and centrifuged at 13,000 rpm. After discarding the supernatant, the column was rinsed once with 100% isopropanol followed by a wash with PB buffer. A final elution was performed by loading 50 µl of EB buffer (10 mM Tris.Cl, pH 8.5) centrifuged at 13,000 rpm into a clean, sterile 1.5 ml Eppendorf tube.

### Cell culturing

Single colony isolates from frozen stock following 24-48 h growth at 30°C were used to inoculate 5 ml liquid culture of either Yeast Extract-Peptone-Dextrose media (YPD: 2% glucose, 1% yeast extract, 2% bacto-peptone) or synthetic drop-out media lacking uracil (2% glucose, 1x yeast nitrogen base; 1x amino acid and base drop-out compositions (SD -URA, Formedium Ltd, UK), according to cell strains and selection requirements. Yeast cells were grown in the prepared liquid culture to mid-log phase (OD_600_ = 0.4-0.6) at 30°C before harvesting for imaging. A 100 mM copper sulphate stock solution was prepared, filter-sterilised with 0.22 µm diameter cut-off filters, and stored at room temperature. For the induction experiments, cells were first grown for 1-4 h in media containing 5 µM copper chelator bathocuproine sulfonate (BCS) before washing and incubation in media containing 100 µM copper sulphate to induce expression via the *CUP1* promotor [86]. To promote the formation of aggregates, cells at the log phase were harvested, diluted to approximately OD_600=_ 0.2 and heat shocked for 2 h at either 37°C, 42°C or 30°C (the latter temperature being the control condition). The cells were then harvested and prepared for imaging with confocal microscopy.

### Vacuole labelling

To label vacuoles, 0.8 µM FM4-64 [87] was added to 1 ml of cell culture in YPD-rich media and incubated with shaking for 1 h. Cells were then washed two times with SC media then grown for a further 1 h chase period in SC media lacking dye. After incubation, samples were prepared for imaging.

### Sample preparation for imaging

Imaging was performed in “tunnel” slides [88] using 22x22 mm glass coverslips (No. 1.5 BK7 Menzel-Glazer glass coverslips, Germany). To immobilize cells to the surface, 20 µl of 1 mg/ml Concanavalin A (ConA) was added to the tunnel slide [89]. Excess ConA was rinsed with 200 µl of imaging media before 20 µl of cells were added, incubated for 5 min upside down in a humidified chamber to promote cell adhesion. Finally, any unbound cells were removed by washing with 200 µl of imaging media and sealed with fast-drying nail varnish before loading on the microscope for imaging [90]. Time-lapse experiments were performed in 35 mm glass-bottom dishes (Ibidi GmbH, Germany) with similar ConA coating methods adapted to the dishes support [91]. 300 µl of 1 mg/ml of ConA were added to the dishes and incubated for 5 min then washed three times with sterile water. The dishes were then dried under a laminar flow hood ready for imaging. Typically, mid-log phase cells were diluted to OD_600_ <0.1 before addition to the ConA coated dish and incubated for 5 min at room temperature. The dish was washed two times with imaging media to remove any unbound cells and finally topped with fresh media for imaging.

### Confocal microscopy imaging

Cell strains were excited using 488 nm and 561 nm wavelength lasers on the LSM 880 Zeiss microscopes with a 1.4 NA (Nikon) objective lens. Intensity and gain were optimised and then maintained for each experiment. Green fluorescence (from mEGFP and mNeonGreen fluorophores) was imaged using 2% laser excitation power and red fluorescence (from the mScarlet-I fluorophore) with 1% power to minimise photobleaching. Detector digital gain was set to 1 with a scanning time of 1.23 seconds per frame. Z stack images to generate 3D movies of cells expressing aggregates were acquired with 0.33 µm thick sections across the sample covering 5-6 µm thickness. FM4-64 vacuolar staining [87] was imaged with the 561 nm wavelength laser at 5% laser power using a bandpass emission filter range set to 578-731 nm. Timelapse imaging was performed by acquiring 10 min intervals of 3 μm thick section slices images over 90 min for optimal cytoplasmic volume visualisation during cell division (as described in previous work [92]).

### ImageJ image analysis

Confocal microscopy data were analysed using ImageJ/Fiji software (ImageJ 2.14.0/1.54f/Java 1.8.0_322) to extract fluorescence intensities from pre-defined segmentation outlines. Cell outlines were generated either manually using the ImageJ selection tool or in a semi-automated process using the Cell Magic Wand plugin [93]. Fluorescent foci within each cell were detected using our bespoke ImageJ macro SegSpot allowing for the selection of a threshold method (within the range of inbuilt thresholding functions available in ImageJ) and object detection function within pre-defined cell outlines or regions of interest stored in ImageJ ROI Manager. Finally, pixel intensities and area parameters of the identified foci were extracted and displayed in an output table (See Supplementary Figure 5). Z stack images were visualized with the 3D project inbuilt ImageJ plugin.

### Slimfield microscopy

Preliminary attempts to measure the mobility of iPAR-labelled aggregates using fluorescence recovery after photobleaching (FRAP) were technically challenging due likely to their relatively high diffusion rates. Because of its single-molecule precise detection sensitivity and rapid millisecond sampling capability, we used Slimfield microscopy [61, 66, 67, 77, 94–96] to characterise the iPAR-labelled aggregates in terms of their molecular stoichiometry (defined as number of fluorescent iPAR tags estimated per distinct fluorescent focus detected) and their mobility within the cell cytoplasm (in terms of the effective diffusion coefficient of tracked iPAR foci). This method enables quantification of the spatial dependence of rapid diffusion *in vivo* in ways that more traditional technologies such as fluorescence correlation spectroscopy (FCS) cannot. Cells expressing iPAR were imaged using excitation via an epifluorescence narrowfield laser beam [96] to generate a Slimfield profile with wavelength 488 nm (Obis LS laser) set to 20 mW power at the sample using 1,000-1,500 frames per acquisition at 5 ms per frame sampling time.

Aggregates were produced following the established standard condition, and cells grown to log phase were induced for iPAR expression using 100 µM copper for 2 h including 1 h heat shock at 37 °C. Osmotic stress with 1 M NaCl and 1.5 M sorbitol was applied and compared to the control condition with cells in 50 mM NaPi.

Protein aggregates were tracked using our in-house software platform which could be implemented in both MATLAB [97] and Python [98] modalities, which uses iterative Gaussian fitting [99] to pinpoint the spatial location of tracked fluorescent foci in complex live microbial cells to approximately 40 nm lateral precision and quantifying stoichiometry, copy number and mobility parameters [100]. Stoichiometry was determined by normalising the initial unbleached track intensity with the brightness value estimated for a single iPAR molecule *in situ* in live cells and from *in vitro* experiments on purified iPAR molecules using step-wise photobleaching, then rendering stoichiometry distributions using kernel density estimation analysis [101].

Diffusion coefficients were estimated from the initial gradient of the mean square displacement versus time interval relationship generated for each track [102, 103], assuming the solution environment is purely viscous as opposed to viscoelastic [104].

## Results

### Construction of an inducible monomeric marker for cytoplasmic aggregation in budding yeast cells

The vacuolar hydrolase CPY traffics through the biosynthetic pathway as an inactive precursor before activation in the yeast vacuole [105]. A mutant version of CPY prone to aggregation, denoted CPY* [44], has been used in previous studies as a model to assess protein folding and regulatory control of misfolded proteins [105–108]. The CPY* variant carries a single amino acid substitution of glycine for arginine at position 255 (G255R) near the enzymatic active site (Figure 1A –C). Deletion of the N-terminal signal peptide (∆ss) of CPY inhibits entry to the secretory pathway and consequently the hydrolase mislocalises to the cytoplasm [109]. The ∆ssCPY* mutant, which aggregates in the cytoplasm, serves as a useful marker for protein aggregation [50, 51, 110, 111]. However, the endogenous *PRC1* promotor [112] typically used to induce expression of this aggregate marker is metabolically regulated [54, 55]; therefore, expression, and aggregation, often vary depending on the specific growth and stress conditions resulting in potential difficulties of interpretation.

**Fig 2:**
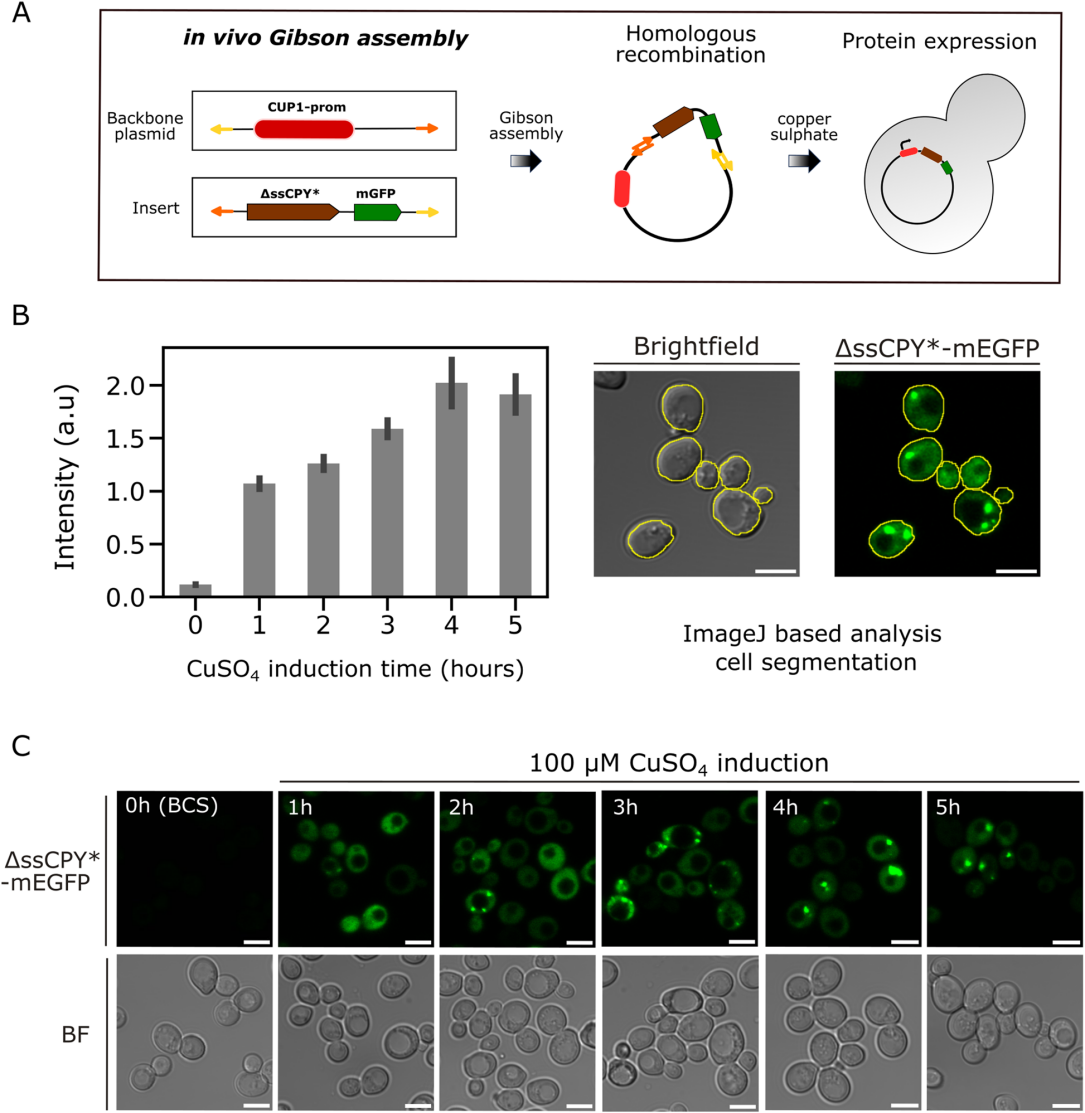
Induction of *CUP1* promoter by copper sulphate results in expression of protein aggregates, visible in confocal microscopy. **A** Schematic representation of cloning strategy to produce copper-inducible cytoplasmic ΔssCPY*-mEGFP aggregates. **B** Bar plot for the fluorescence intensity of *CUP1*-ΔssCPY*-mEGFP incubated in the copper chelator BSC (0 h) or following induction by 100 µM copper sulphate, at 1 h, 2 h, 4 h and 5 h, *n* = 100 cells for each condition, s.e.m. error bars represented. The micrographs on the right show cell segmentation using the Cell Magic Wand ImageJ tool applied to brightfield images. These segmented images were then used to quantify the total fluorescence intensity from the GFP channel corresponding to each cell. **C** Fluorescence micrographs representing the ΔssCPY*-mEGFP aggregation at different induction time points

To overcome this limitation, we generated a fusion construct which expressed ∆ssCPY* from the copper inducible *CUP1* promoter [113] in the presence of 100 µM copper sulphate (see Methods and schematic Figure 2A), using definitive monomeric fluorescent protein tags (monomeric EGFP in the first instance) to mitigate against issues associated with fluorescent protein oligomerization. Using a titration from 0 - 200 µM copper sulphate on a GFP-tagged methionine permease we previously used for membrane trafficking studies [81]. We have routinely used the *CUP1* promoter because under basal media conditions, which have a very small amount of copper, expression levels are low. Although expression can be further reduced with copper chelation, this also inhibits cellular growth [114]. Therefore, we use media lacking copper to culture cells to appropriate log phase and density for experiments, before adding up to 100 µM copper to robustly induce expression. However, 100 µM copper has no detectable phenotype on cellular process we have measured. Furthermore, we confirmed that copper had no measurable effect on fluorescence levels. Flow cytometry was used to define background fluorescence in wild-type cells and distinguish fluorescence of Mup1-EGFP expressing cells (Supplementary Figures 1 and 2).

Copper-dependent expression levels of *CUP1*-∆ssCPY*-mEGFP in budding yeast cells were characterised using confocal microscopy. Induction times from 1 - 5 h were used followed by imaging and subsequent image segmentation analysis to extract the fluorescence intensity and the integrated pixel volume information of cells and protein aggregates. We found that expression of iPAR could be rapidly induced in the presence of 100 µM copper sulphate (Figure 2B), with a strong increase observed after 1 h copper exposure, with an apparent slowing after 2 h and 3 h exposure and steady-state expression levels after approximately 4 h (Figure 2B and C). At 5 h induction we noticed a small decrease in fluorescence intensities, which was consistent with the activity of clearance pathways associated with protein aggregation.

A 2 h copper incubation time was selected as a standard induction condition to express the ΔssCPY*-mEGFP marker to generate a sufficient pool of protein aggregates for subsequent analysis. We noticed that after 2 h expression there was a reasonable level of expression and several aggregates forming in the cytoplasm (Figure 2C).

**Fig 3:**
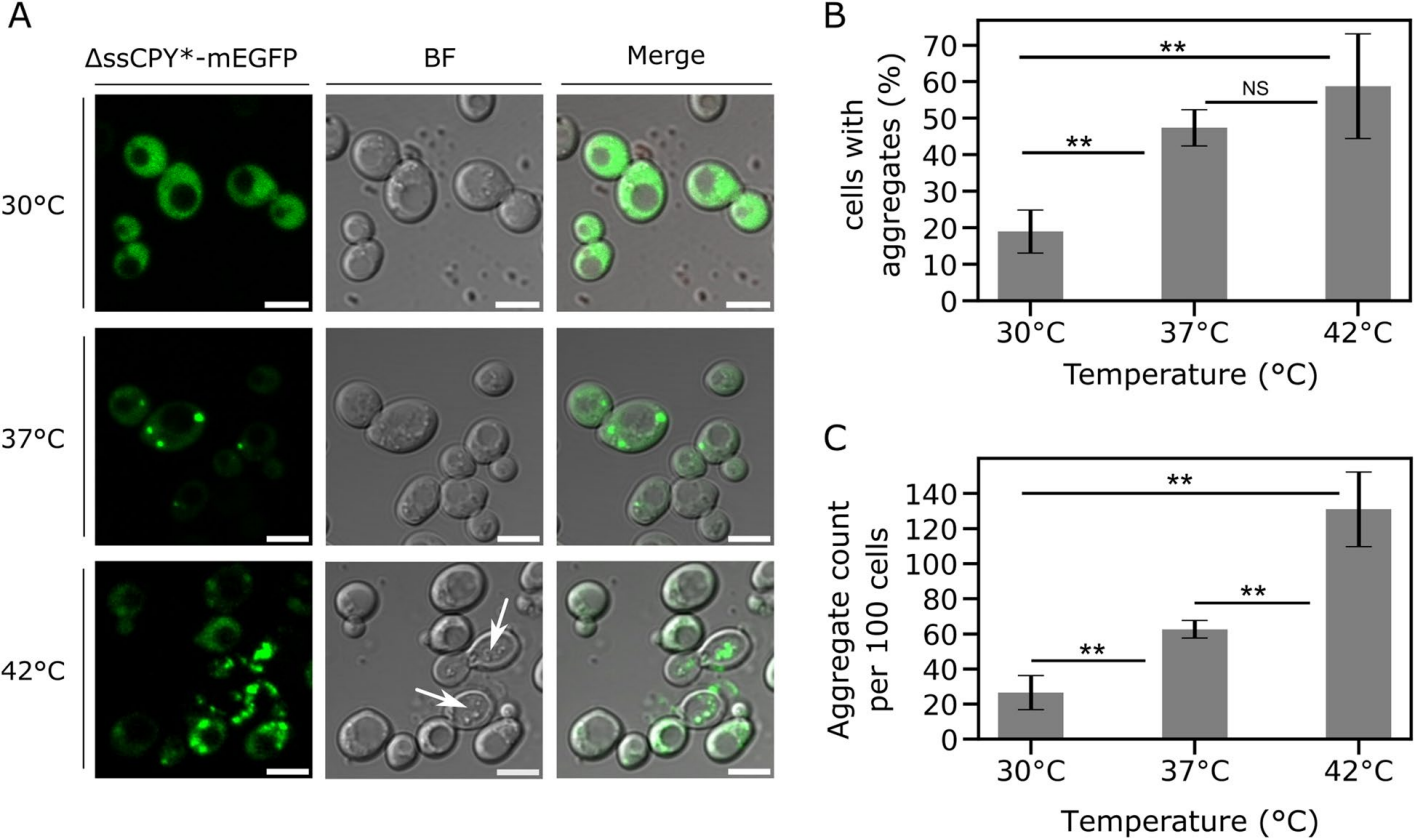
Short-term heat shock induces the formation of aggregates. **A** Confocal micrographs from a representative cell population of yeast cells expressing the *CUP1*-ΔssCPY*-mEGFP protein after induction with copper sulphate for 2 h followed by 1 h at either the initial growth temperature 30°C or the heat shock temperatures of 37°C and 42°C. White arrows indicate dead cells in the brightfield channel, which were not used in subsequent analysis. Scale bar: 5 µm. **B** Bar plot representing the percentage of cells which were positive for aggregates for cells exposed to the control 30°C, or the 37°C and 42°C heat shock. Non-significance is indicated by a Student’s *t*-test *p* value ≥0.05, the double asterisk indicates a *p* value <0.05. **C** Bar plot showing the number of aggregates detected and counted in the cell population, bringing it to *n* = 100 cells in total, s.d. = error bars. See also Supplementary Table 2

We then characterised the effect of temperature on cells expressing ΔssCPY*-mEGFP following heat shock. As expected, cells grown for 1 h at 30°C exhibited very few protein aggregates, however, shifts to heat stress conditions using temperatures of 37°C or 42°C resulted in measurable iPAR aggregate formation (Figure 3A). There was a significant increase in cells following heat shock at both 37°C or 42°C in comparison to any cells at 30°C that had detectable aggregates of ΔssCPY*-mEGFP (Figure 3B). A significant increase in number of aggregates was observed, in addition to the number of cells in which aggregates were detected, following heat stress (Figure 3C).

Between 30°C and 37°C, we observed an increased number of aggregate-positive cells (defined as a cell which contains at least one detected iPAR fluorescent focus) by a factor of approximately 2.5, from an average of 19% (±5.8, s.d.) to 47% (±9.4), corresponding to a Student’s *t*-test *p* value of 7.59 x 10^-5^ (i.e., highly significant). Similarly, between 30°C and 42°C, the pool of aggregate-positive cells increased by a factor of approximately 3 from 19% (±5.8) at 30°C to 59% (±14.3) at 42°C with a significant *p* value of 5.00 x 10^-3^. Although 42°C induced a greater number of aggregate foci across the population, we also detected elevated levels of cell death (Figure 3A; arrows). Additionally, there was no significant increase in aggregate-positive cells by heat shocking at 42°C compared with 37°C (*p* = 0.261) (see Figure 3B and Supplementary Table 1).

The total number of detected aggregates increased by a factor of 2.4 from 30°C to 37°C, and by a factor of 4.9 between 30°C and 42°C; and, although the number of aggregate-positive cells was similar between 37°C and 42°C, we still observed a significant increase in the number of aggregates detected (Figures 3B,C and Supplementary Table 2). We subsequently used 2 h copper induction followed by 1 h heat shock at 37°C as our standard protocol, which we found to be sufficient to induce trackable ΔssCPY*-mEGFP aggregates without compromising the phenotype or viability of the cells.

**Fig 4:**
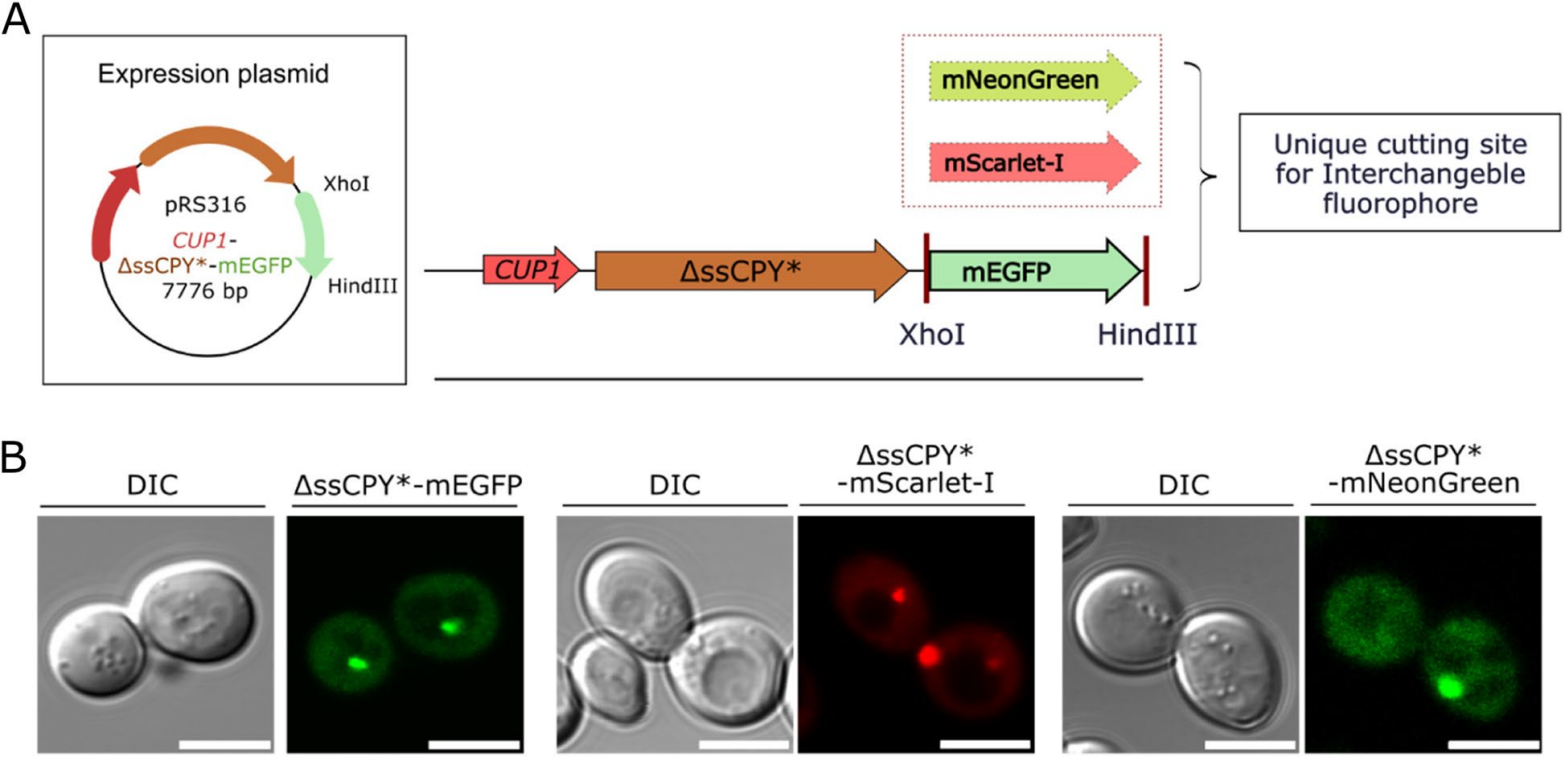
iPAR enables interchangeable monomeric fluorescent proteins to be used for reporting on protein aggregation inside the cytoplasm of living yeast cells. **A** Schematic of the expression plasmid constructed for *CUP1*-ΔssCPY*-mEGFP, the fluorophore with *Hind*III and *Xho*I cutting sites used to facilitate the exchange of fluorescent markers. **B** From left to right, micrographs with differential interference contrast (DIC) and fluorescence channel for *CUP1*-ΔssCPY* in pRS316 with the mEGFP, mScarlet-I and mNeonGreen fluorescent proteins shown respectively

To expand the utility of the iPAR reagent, the mEGFP fluorescent tag was flanked with unique cutting sites (5’ *Hind*III and 3’ *Xho*I sites) to enable interchangeability and future extension of the construct library for DNA insertion to encode different fluorescent proteins (Figure 4A). We used this strategy to create iPAR variant *CUP1*-ΔssCPY*-mNeonGreen and *CUP1*-ΔssCPY*-mScarlet-I, which we found also formed inducible aggregates following the optimised protocol described above in a qualitatively similar manner (Figure 4B).

### Cytoplasmic aggregates and localisation in time and space in budding yeast

**Fig 5:**
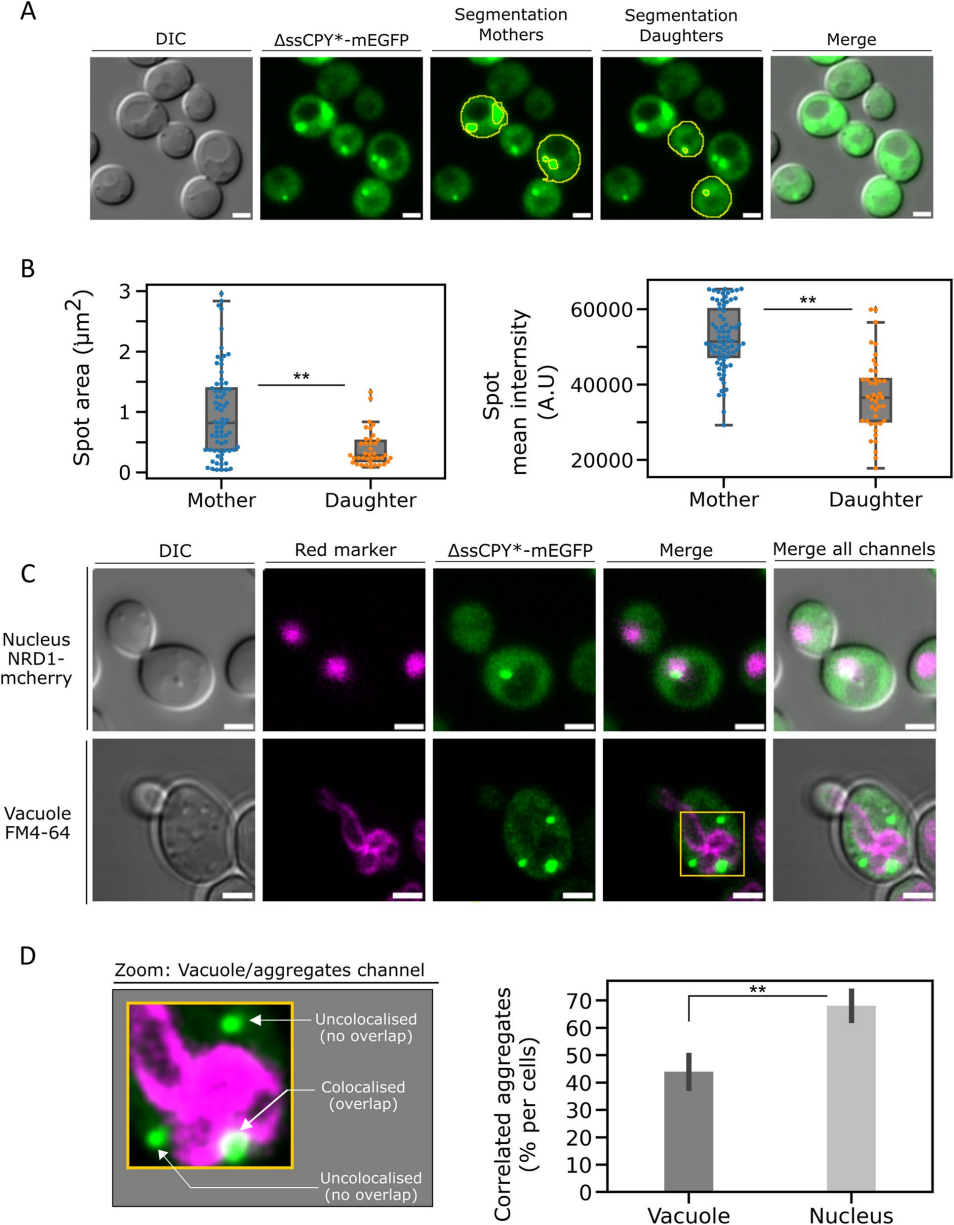
Protein aggregates localise specifically to vacuolar and nuclear compartments. **A** Semi-automated segmentation (a combination of the ImageJ selection tool and our bespoke automated macro processing) of mother cells and daughter cells to characterize fluorescent foci. From left to right: DIC image of the cell, fluorescence channel, segmentation of the mother cells, of the daughter cells and merge of the fluorescence channel with the DIC. Scale bar: 2 µm. **B** Characterization of aggregate foci, jitter plot of the detected foci area between mother cell and daughter cells. On the right, jitter plot of the intensity measured in each fluorescent focus identified. Outlier detection and removal was performed using standard interquartile methods [115, 116]. **C** Fluorescence micrographs of dual label strain for simultaneous observation of aggregates and key cellular compartments. Top row shows the nucleus labelled by nuclear reporter Nrd1-mCherry background strain, bottom row shows the vacuole labelled with FM4-64 [87], which mark the vacuole location. Micrographs showing the brightfield, the red channel with the marked compartment of interest, the green channel with the iPAR aggregate reporter and the merge of both fluorescence channels along the brightfield. Scale bar: 5 µm. **D** (left) Zoom-in of region highlighted in panel C with (right) estimate of the percentage of detected aggregates which are either colocalised with the vacuole or nucleus compartments (s.d. errorbars, *n*=100 cells, the double asterisk indicates a *p* value <0.05)

We performed further characterisation of iPAR to focus on spatiotemporal dynamics of newly formed aggregates. We first investigated the number of aggregates and their spatial distributions between mother and daughter cells. Figure 5A shows the analysis focused on budding cells, where mother and daughter cell images were independently segmented using our bespoke SegSpot macro coded for ImageJ which enabled thresholding and object detection of fluorescent foci (see Methods and Supplementary Figure 4). The area and intensity of fluorescent foci were automatically extracted by this macro, and their values plotted (Figure 5B). Jitter plots revealed that the mean foci areas measured in mother cells were approximatively twice as large as those measured in daughter cells, with a mean focus area of 0.99 (±0.74) µm^2^ measured in mother cells v*s* 0.39 (±0.29) µm^2^ for daughters (Figure 5B: left plot and Supplementary Table 3). Mother cells contained aggregates of higher volume with a mean fluorescence intensity significantly higher than daughter cells, corresponding to an integrated intensity (measured in arbitrary units A.U., rounded to nearest 100 A.U.) of 52,400 A.U. (±8,500) *vs* 37, 100 A.U. (±9,800) respectively (right plot of Figure 5B, see also Supplementary Table 3). We note that the distribution of numbers of aggregate foci in both cell types is heterogeneous but more pronounced in mother cells (Figure 5B), which was also reflected by higher standard deviation values. These results suggest a polarity behaviour of formation/clearance of ΔssCPY*-mEGFP during cellular growth resulting in statistically different sizes of aggregates between two cells which are dividing (the older cells displaying larger aggregates with higher intensities than those of the emerging daughter buds).

We then sought to verify whether iPAR indicated any qualitatively similar spatiotemporal behaviour as reported previously for other cytoplasmic aggregation reporters [53, 117, 118]. For example, ΔssCPY* aggregates were previously shown to be localized in JUNQ and IPOD [119] inclusion bodies, observed near the nucleus [120] and the vacuole [121], respectively. To elucidate whether our induced ΔssCPY*-mEGFP colocalised near the membrane of either of the nucleus or the vacuole, we constructed dual colour cell strains including a fluorescent red tag as a reporter for the location of the nucleus or the vacuole. Figure 5C shows the resulting dual colour images of representative live cells, the top row showing Nrd1-mCherry [59, 122] marking the nucleus, the bottom row showing using FM4-64 pulse-chased labelling to mark the vacuole (see Methods), both simultaneously expressed with ΔssCPY*-mEGFP.

**Fig 6:**
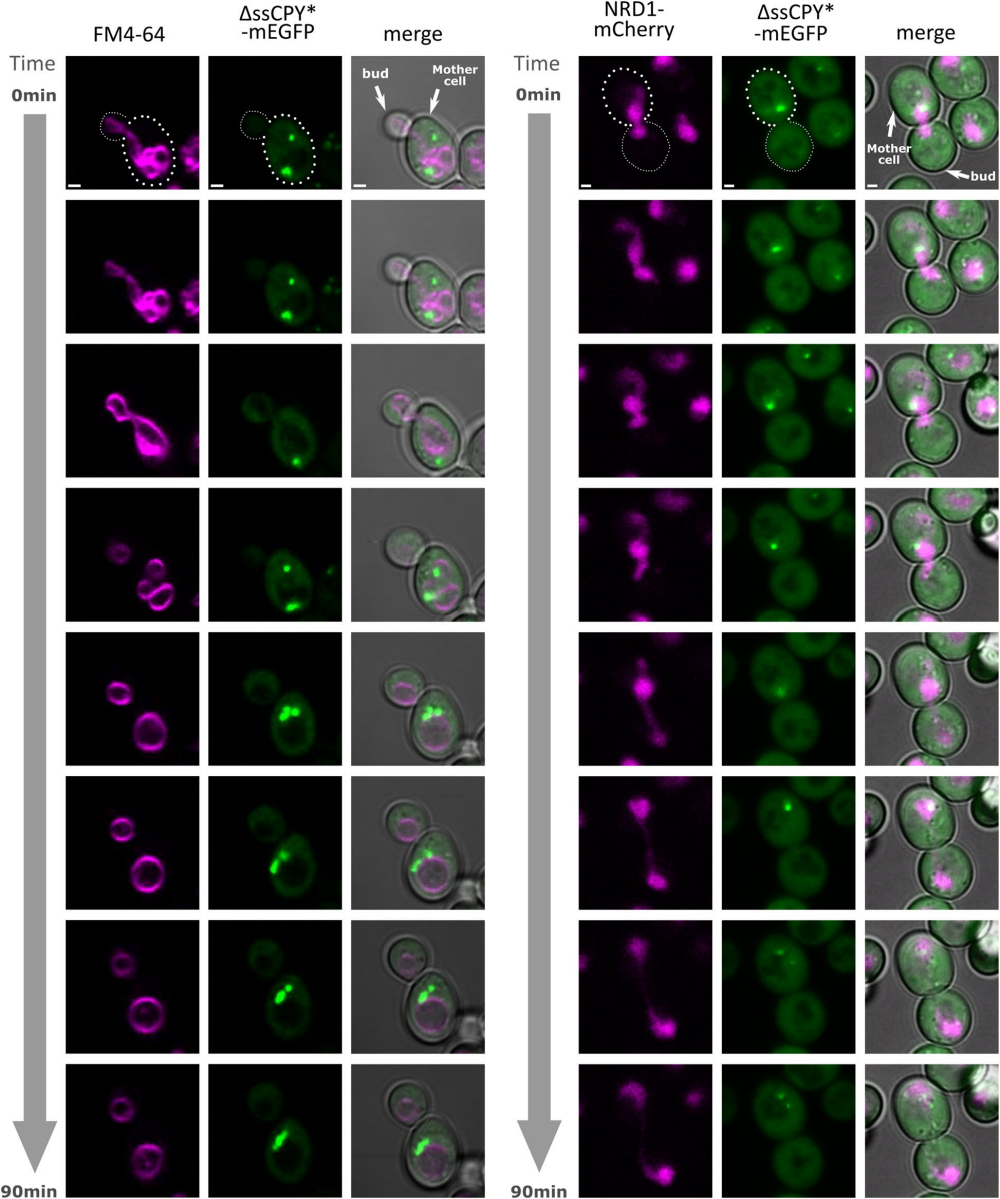
Protein aggregates are localized near to the vacuole and nucleus during cell division. Cells expressing the ΔssCPY*-mEGFP trackable aggregates (generated after 2 h copper sulphate induction including 1 h heat shock at 37°C) in combination with either Nrd1-mCherry expressed in the nucleus or a WT background strain labelled with FM4-64 [87] at the vacuole, imaged using confocal microscopy over 90 min during cell division. Micrographs show the red channel for those two markers of interest, the green channel of the imaged aggregate marker and the merge of both fluorescence channels along the brightfield. White arrows indicate the mother cell and the bud position. Scale bar: 1 µm

We quantified the proportion of aggregates present in each cellular compartment, by assessing the proximity/colocalization of both colours (micrographs in Figure 6D) and found that a mean of approximately 44% of aggregates colocalised with the vacuole compartment and 68% with the nucleus (Figure 5D and Supplementary Table 4). This result is broadly consistent with earlier observations that a significant number of aggregates appear to localise both near the nucleus or vacuole [93]. The higher percentage of aggregates identified as being associated with the nucleus may indicate that aggregates preferentially sequestrate into JUNQ inclusion bodies.

We also acquired 3D data to visualise the patterns of aggregates spatial expressions inside the entire volume of the cell (Supplementary Figure 3 and Supplementary Videos 3-6). 3D projections of cells expressing iPAR, including labels of either the vacuole or nucleus, further confirmed the presence of cytoplasmic aggregates, appearing preferentially in the mother cells and confirming localisation in regions that are in likely contact with the nucleus and vacuole membrane to within our optical resolution limit of approximately 250 nm.

Finally, we performed time-course experiments during cell division with the dual label strains detailed above. In both cases, as a cell divides, we observed protein aggregates sequestrated in the mother cell (Figure 6 and Supplementary Videos 1 and 2). We observed that both vacuoles and nuclei were inherited into budding daughter cells whilst aggregates were retained in the mother cells. We note both events occurs at different stage of the cell cycle, the vacuole is inherited at early stages (~20 min) of the budding process but the nucleus is one of the last (~60 min) [123].

This observation reinforces the hypothesis that there is a diffusion barrier between mother and daughter cells maintained during cell division [123–125]. The sequestration of misfolded cytoplasmic proteins has been reported previously as being a highly conserved quality control process which is crucial to cellular rejuvenation [126–130]; the presence of ΔssCPY* associated with both JUNQ and IPOD inclusion bodies suggests a potential cellular recognition and cellular response for clearance and degradation.

### Using iPAR in conjunction with Slimfield to quantify the molecular stoichiometry of aggregates and their spatial distribution and mobility in live cells

**Fig 7:**
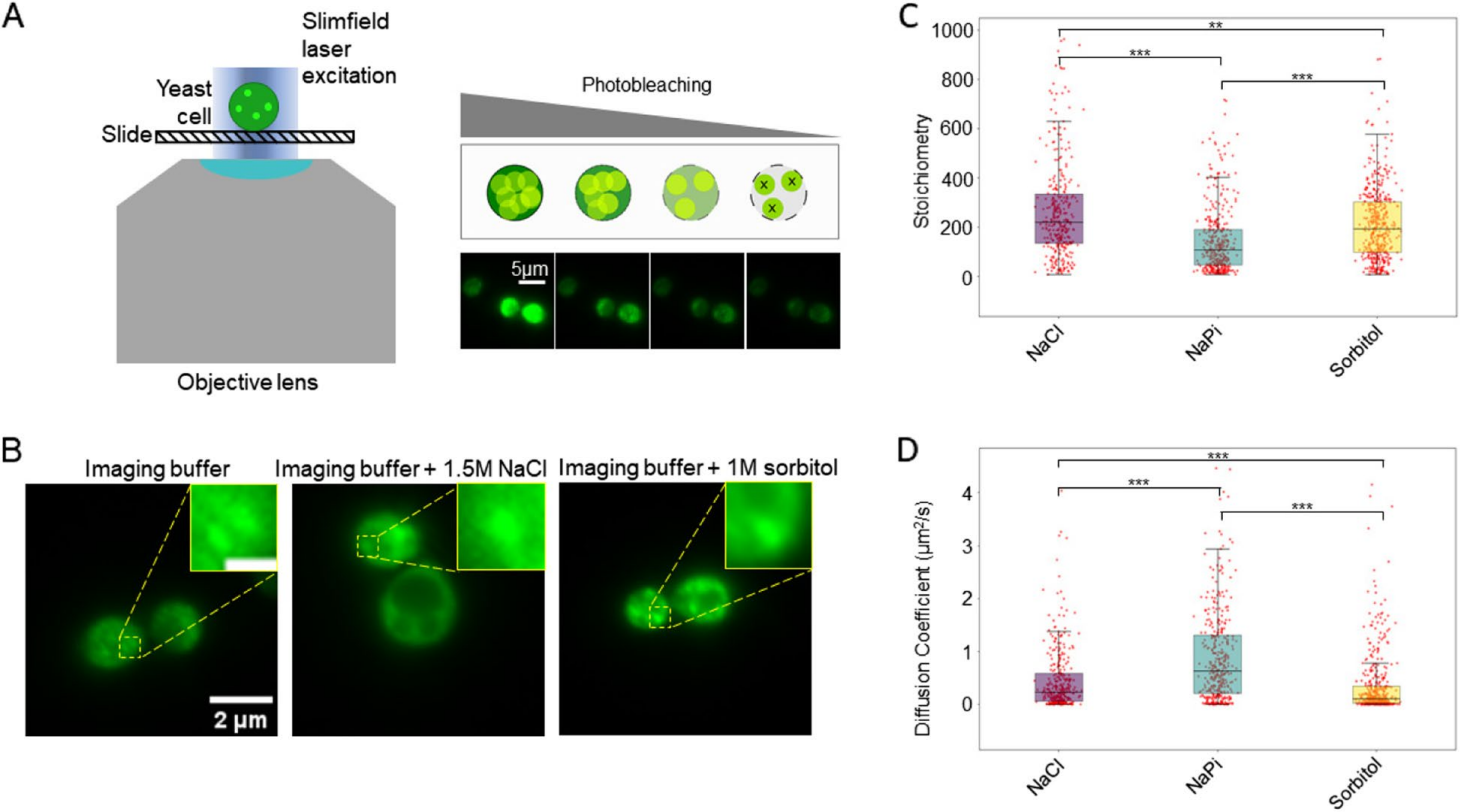
iPAR labelling is compatible with single-molecule precise millisecond timescale Slimfield microscopy. **A** (left) cartoon representation of Slimfield excitation, in which the width of the laser beam is only a little larger than the diameter of a single cell, utilising the associated increased laser excitation intensity to enable detection of single iPAR molecules above the camera detector noise; (right) schematic representation of photobleaching of iPAR molecules inside cells which enables the detection of single molecules due to the subsequent increased mean spatial separation of remaining unbleached iPAR molecules, also visualised using yeast expressing ΔssCPY*-mEGFP. **B** Representative images of yeast expressing ΔssCPY*-mGFP in normal imaging buffer, or under hyperosmotic stress in the form of 1.5 M NaCl or 1 M sorbitol respectively, insets showing distinct aggregate foci (inset scale bar 500 nm). **C** Comparison of aggregate stoichiometries, and (**D**) diffusion coefficients, under the previously mentioned stress conditions using box plots indicating the median value and interquartile range, with the aggregate populations in each condition showing statistically significant differences when compared using a Mann Whitney U test (for stoichiometries the corresponding *p* values (1 d.p.) are: NaCl:NaPi=1.1 x 10^-23^, NaCl:sorbitol=1.1 x 10^-3^, NaPi:sorbitol=1.4 x 10^-14^; for diffusion coefficients the corresponding *p* values are: NaCl:NaPi=3.3 x 10^-14^, NaCl:sorbitol=7.0 x 10^-8^, NaPi:sorbitol=7.3 x 10^-33^). Number of tracked foci for NaCl *n*=337, NaPi *n*=393, sorbitol *n*=430

We used Slimfield on live cells expressing the mEGFP iPAR variant to enable us to the count how many iPAR molecules are present in aggregates and how rapidly aggregates diffuse inside cells (Figure 7A). Cells were visualised in normal 50 mM NaPi imaging buffer, as well as 50 mM NaPi supplemented with either 1.5 M NaCl or 1 M sorbitol, typical conditions to induce hyperosmotic stress; NaCl and sorbitol are both crowding agents of distinct nature therefore with a different potential of interaction on metabolic functions and oligomerisation [131].

Slimfield images exhibited distinct fluorescent foci corresponding to protein aggregates (Figure 7B), qualitatively similar in appearance to those observed with confocal and epifluorescence microscopy, which could be pinpointed using our bespoke localisation microscopy tracking software, optimised in budding yeast cells to a lateral spatial precision of approximately 40 nm [132]. This analysis software enabled measurement of molecular stoichiometry of each tracked aggregate by using a method which converts their quantified integrated pixel brightness into the number of photoactive iPAR molecules utilising a stepwise photobleaching protocol [19] to determine the brightness of a single fluorescent protein molecule [88].

We observed an increase in stoichiometry for both of the hyperosmotic stress conditions applied, from a mean of 157 (±25) molecules per aggregate for the non-stress condition with cells in 50 mM NaPi buffer to 290 (±28) for 1 M NaCl (osmolarity equal to 2 osmol/L) corresponding to an 85% increase, while the stoichiometry measured for 1.5 M sorbitol (osmolarity equal to 1.5 osmol/L) was 217 (±17), a 38% increase compared to the control condition (Figure 7C). The tracking software also enabled estimates of the lateral diffusion coefficient for each aggregate, indicating an associated reduction of aggregate mobilities in a hyperosmotic extracellular environment, consistent with an associated increase in intracellular molecular crowding [92]. The control condition shows a diffusion coefficient of 0.99 (±0.15) µm^2^/s compared to 0.47 (±0.04) for 1 M NaCl and 0.36 (±0.06) µm^2^/s for 1.5 M sorbitol, corresponding to a decrease of 48% and 36% respectively. This quantitative analysis exemplifies iPAR being used in conjunction with an example of rapid single-molecule bioimaging tecnhology, Slimfield. It robustly quantifies differences of aggregation due to different hyperosmotic stress factors, for example the effect on aggregate stoichiometry and diffusion is of a greater extent when induced by 1 M NaCl salt exposure than for 1 M sorbitol, consistent with simple colligative differences is osmolarity.

It reveals a broad range for both stoichiometry and diffusion coefficient for aggregates, an observation which resonates with the concept of aggregate formation being driven by dynamic and heterogeneous protein nucleation inside cells. These observations indicate that these extracellular hyperosmotic environments bias the likelihood of protein nucleation events that result in aggregate formation.

More generally, these findings show that iPAR is compatible with high-precision rapid single-molecule localization microscopy using different osmotic stress factors to study protein aggregation in live cells.

## Discussion

We have developed iPAR, an improved reporter for high-precision quantification of cytoplasmic protein aggregation in the budding yeast *S. cerevisiae*. By replacing the metabolically regulated *PRC1* promoter with the copper sulphate inducible *CUP1* promoter and introducing definitively monomeric fluorescent tags, iPAR enables precise control of protein expression in growing cells with reduced interference from the fluorescent tag in the aggregation process. These modifications offer an alternative choice of reporter for stress-related studies and for investigating the dynamics of protein aggregation, compared to heat shock protein biomarkers of aggregation which use non-monomeric GFP [115]. As with all fluorescent protein probes the mEGFP used in iPAR will have a maturation time. In the context of the experiments described here ΔssCPY*-mEGFP is expressed for 2 hours before imaging which provides ample time for maturation of mEGFP (~22 mins [59, 88], however this maturation time could prove limiting in experiments that require the immediate imaging of iPAR upon expression. It should also be noted that any stoichiometry measurements of ΔssCPY*-mEGFP within aggregates are likely to be an under representation (in the region of 7% [59]) of the true number due to the presence of dark constructs that are non-photoactive. As proof-of-concept, we used 1M NaCl and 1.5M sorbitol to induce different levels of cellular hyperosmolarity, though interesting future work could titrate these respective levels to compare phenotypic responses of these different osmolytes but at comparable osmolarities.

We first characterised iPAR by measuring the expression response of ΔssCPY*-mEGFP to 100 µM copper sulphate, indicating that a 2 h standard induction time was optimal to produce a strong fluorescence signal of protein aggregates. We then tested the effects of heat shock on aggregation following inducible expression. At 37°C, we measured a strong increase in aggregate-positive cells (greater than twice as many cells that contain protein aggregates compared to cells incubated at the 30°C no-stress control condition). At 42°C, we observed a similar number of aggregate-positive cells, but we detected a higher total number of aggregates across a population of cells as well as a higher number of aggregates per cell. However, the physiological cell phenotype of 42°C was visibly impaired in several instances, including abnormal morphology and dead cells, consistent with cell metabolic malfunction resulting in an increase in cytoplasmic aggregation. Therefore, we did not select this temperature in subsequent investigations using iPAR. A concentration of 100 µM copper sulphate was sufficient to induce aggregate formation and not to generate cellular defects from copper toxicity; future work in titrating different concentration levels of copper sulphate and observing function responses regarding aggregate properties could be valuable.

We verified that induced aggregates localise to the nucleus and vacuole JUNQ and IPOD compartments respectively, as reported from previous studies using existing aggregation reporters. We performed time lapse confocal microscopy imaging to quantify the extent of inheritance of the vacuoles and nuclei during asymmetric cell division of iPAR yeast cells in real time, showing directly on a cell-by-cell basis that these intracellular organelles are inherited to daughter cells whilst proteotoxic aggregates are retained in the mother cell (see Figure 6 and Supplementary Videos 1 and 2). These time-resolved observations taken using the same individual cells are consistent with earlier reports using separate imaging of organelles and aggregates across several different cells [118, 126], however, this is to our knowledge the first direct observation that such aggregates which appear to be associated with specific organelles are, unlike the organelles themselves, not inherited.

In budding yeast cells, the presence of multiple inclusion bodies typically observed during osmotic stress were shown previously to be further sequestrated in targeted cellular locations [118, 133]. Aggregates may be actively recognized by cells and sequestrated in the mother cell volume, additionally, physicochemical properties such as local viscosity [134] and the molecular crowding at the junction between the two cells can potentially influence aggregate localisation, as suggested by the results of our previous study [92] on the investigation of sub-cellular crowding dynamics. This molecular crowding at the junction between two cells may hold a key as to why these toxic aggregates are not inherited alongside their associated organelles. Experiments utilising iPAR with high-precision Slimfield measurements probing this putative junction effect may be valuable future experiments to address this hypothesis since, as we demonstrate here, Slimfield has the capability to robustly quantify the spatiotemporal dynamics of iPAR aggregates, showing that they are mobile inside cells and are comprised from as few as a few tens of molecules up to several hundred, whose mean value increases with extracellular hyperosmotic stress.

Slimfied microscopy enables rapid tracking of aggregates over the entire cell, provided they are within the ca. 1 micron depth-of-field of the microscope and have sufficient contrast against background noise for detection. This enables measurement of the spatial dependence of rapid molecular mobility. Other measurement approaches for quantifying molecular mobility could in principle be used, for example FRAP and FCS, however the relatively slow scanning speeds currently prohibit easily reproducible measurements of molecular mobilities in different regions of the same cell at the same point in time. There are also a suite of different fluorescent-based super-resolved single-particle tracking approaches which could be used in complement to Slimfield [135, 136], though unless specific efforts are made to adapt these the sampling times which are possible are slower than Slimfield’s rapid sub-ms capabilities. It should also be noted, that Slimfield uses a localization microscopy approach which can pinpoint single molecular assemblies that span effective diameters from a few nm up to several hundred nm to a spatial precision which is an order of magnitude better than the optical resolution limit; it is *de facto* a super-resolution method. One interesting route for future study could be to use iPAR labelling to explore the effect of chirality on protein assembly processes, for example as is seen in several filamentous biopolymers [137], and even more generally to study “single-molecule cell biology” [138] in the context of “single-molecule cellular biophysics” [139] such as the soft matter properties of cellular material at a single-molecule precise level [140], e.g. stress relaxation effects [104].

There are a range of approaches which have been developed for reporting on aggregate formation in cells. For example, previous studies reporting aggregation of alpha-synuclein amyloid filaments include light-inducible protein clustering system for *in vivo* analysis [141] and multidimensional imaging tools such as and fluorescence lifetime imaging (FLIM) and super-resolution methods such as structured illumination microscopy (SIM) [142] as well as stepwise photobleaching to assess the number of protein subunits present [19]. Also, studies involving aggregation effects more generally as stress responses seen in “aggresomes” both in yeast [143] and in bacteria [9, 144].

Although maturation effects of the fluorescent proteins we use here are unlikely to account for more than 10-15% of “dark” fluorescent protein in unstressed cells [59, 88], there are potential physicochemical limitations which may need to be considered. For example, issues with tag folding or localization specifically in high-stress conditions. Similarly, there made be issues relating to spatial variation of pH and molecular crowding, and differences relating to the effects of fluorescent proteins on biomolecular liquid-liquid phase separation [68], which may merit future investigation.

In summary, iPAR offers a robust and improved capability to report on cytoplasmic protein aggregation and shows promising potential to offer new insights into the roles played by stress factors in influencing protein aggregation. We have made the plasmids that encode three fluorescently-tagged variants openly available as a research resource to the scientific community to, we hope, contribute to a wide range of future scientific studies, applicable to a range of advanced fluorescence microscopy modalities [145] including advancing single-molecule biophysics approaches [146, 147] as well as aiding new understanding to the soft matter physics rules behind protein aggregation [71, 140]. More generally, our new iPAR technology, has potential to be adapted to other eukaryotic model systems. However, we are careful not to overstate any of the observations we make here in budding yeast in being directly relevant to human cells. Significant additional optimisation is likely to be required to take the iPAR system we have developed here into a human cellular environment if the aim is to directly assess pathology. But with such adaptations there is certainly potential to address several relevant ageing studies and diseases in which protein aggregation is a known or hypothesised factor.

## Supplementary Information

Supplementary Material 1.

Supplementary Material 2.

Supplementary Material 3.

Supplementary Material 4.

Supplementary Material 5.

Supplementary Material 6.

Supplementary Material 7.

## Data Availability

Raw data can be openly accessible from Zenodo, DOI: 10.5281/zenodo.10468170 (https://zenodo.org/records/10468171). Segmentation analysis code can be openly accessible from https://github.com/york-biophysics/ImageJ-Macros (file name: SegSpot.ijm). The Plasmid construct and cloning maps presented in this article were submitted to Addgene genomic bank under the following ID: 83606 (catalogue number: #212197) and accessible online from https://www.addgene.org/212197/. Segmentation analysis code can be openly accessible from https://github.com/york-biophysics/ImageJ-Macros (file name: SegSpot.ijm). The Plasmid construct and cloning maps presented in this article were submitted to Addgene genomic bank under the following ID: 83606 (catalogue number: #212197) and accessible online from https://www.addgene.org/212197/.
