# Supplementary material for: iPAR: a new reporter for eukaryotic cytoplasmic protein aggregation
Source: BMC Methods. 2025 Apr 1;2(1):5. doi: 10.1186/s44330-025-00023-w (PMC11958454; doi:10.1186/s44330-025-00023-w)
Supplement: Supplementary file 1 — Supplementary Material 1. [file 44330_2025_23_MOESM1_ESM.pdf]

Supplementary Information

iPAR: a new reporter for eukaryotic cytoplasmic protein aggregation

Sarah Lecinski[1], Jamieson A.L. Howard[1], Chris MacDonald[2,3], Mark C. Leake[1,2,3,4]

[1] School of Physics, Engineering and Technology, University of York, York, YO10 5DD, UK.

[2] Department of Biology, University of York, York, YO10 5DD, UK.

[3] York Biomedical Research Institute, University of York, York, YO10 5DD, UK.

[4] Correspondence to mark.leake@york.ac.uk

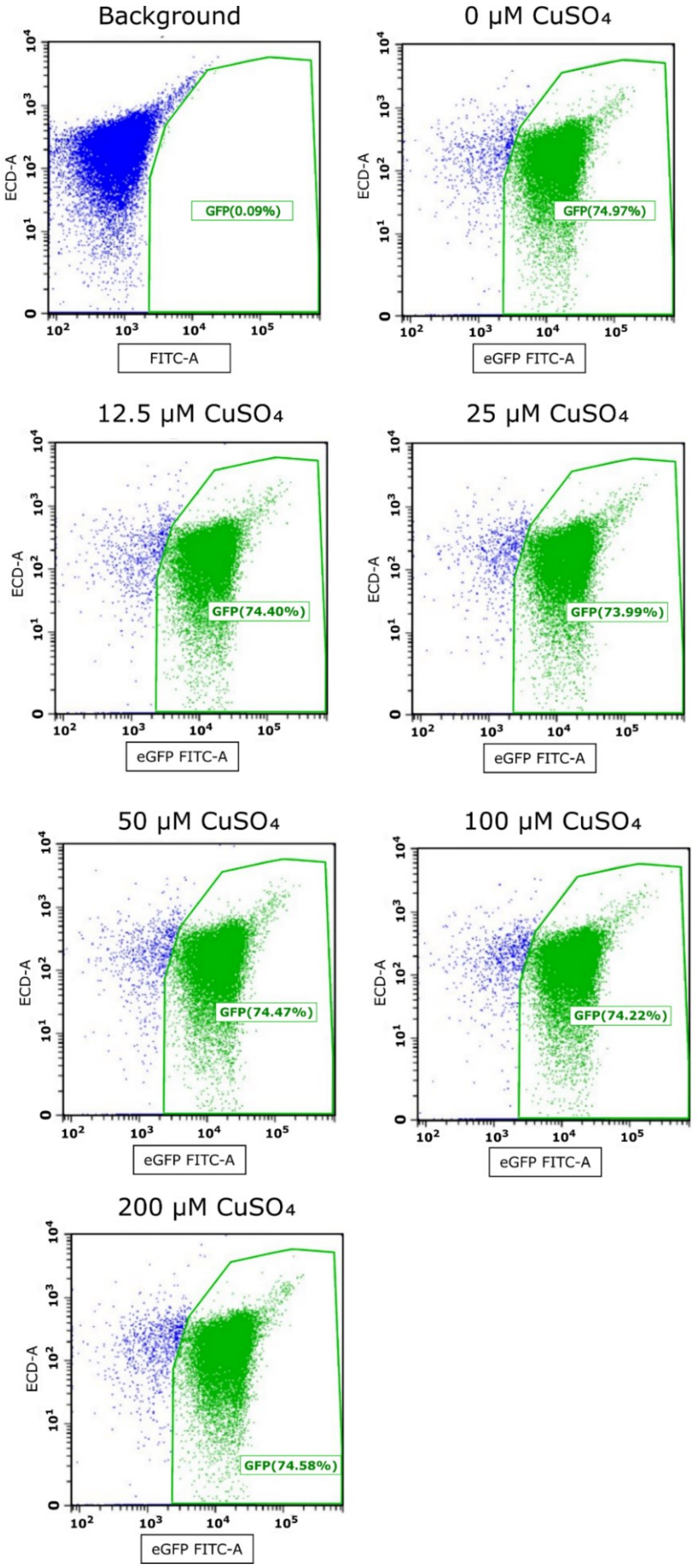

Supplementary Figure 1: Flow cytometry can quantify fluorescence of cells with high throughput, following induction by copper sulphate. Scatter plot representing the presence of

fluorescent positive cells in the cell population analysed by flow cytometry. The background non-fluorescent strain was used to calibrate the presence of non-fluorescent cells, in blue colour. Positive cells expressing Mup1-EGFP are visualised in green and the calculated percentage of EGFP positive is indicated in green.

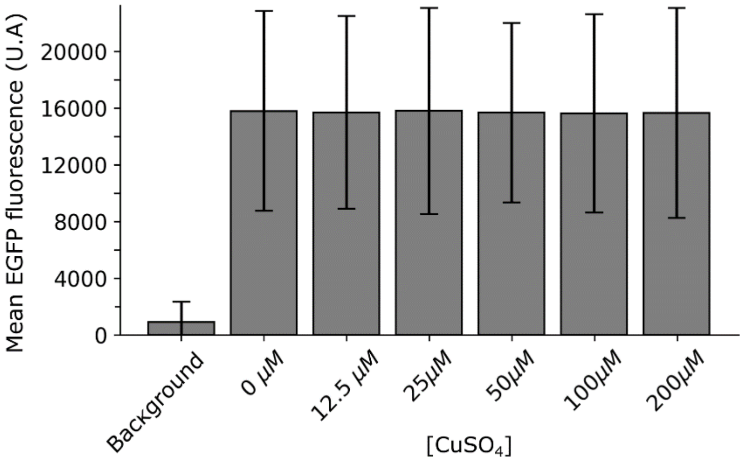

Supplementary Figure 2: Flow cytometry indicates relative insensitivity to fluorescence brightness for different concentrations of copper sulphate. Box plot representing the mean fluorescence measured in cell population expressing Mup1-EGFP in the presence of different copper sulphate concentrations. Error bar SEM. Number of cells n =>10^4.

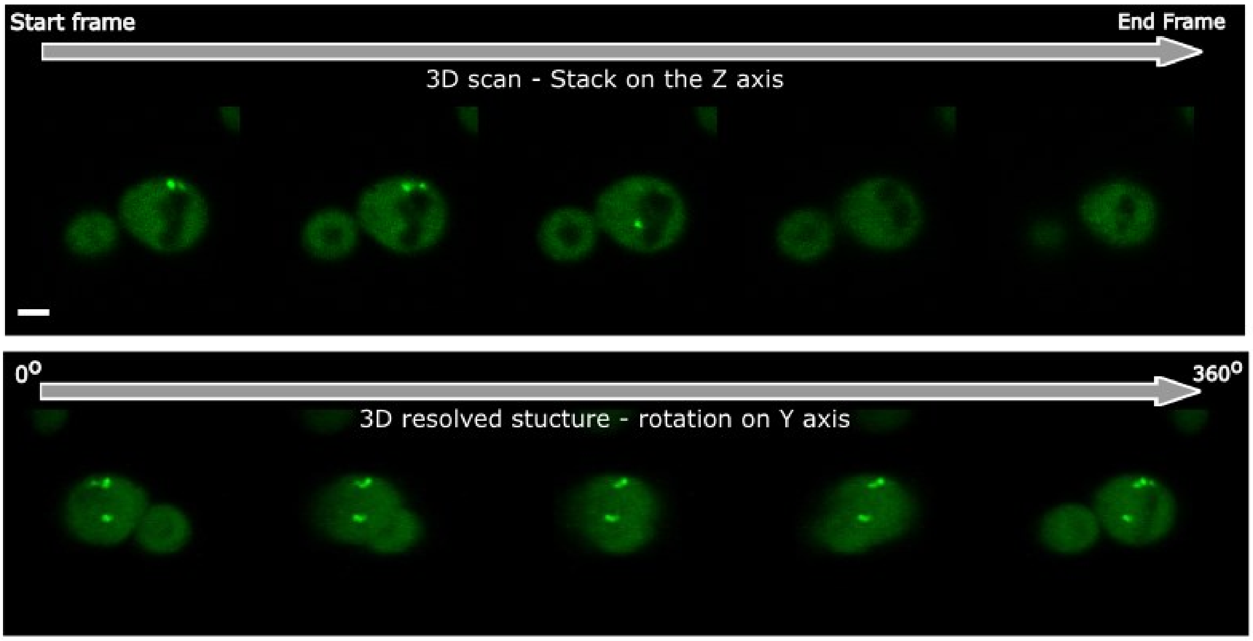

Supplementary Figure 3: 3D visualization can be used to determine the localisation of protein aggregates throughout the full cell volume. Top micrograph: Z stack of protein aggregates for iPAR reporter using mEGFP, 0.33 µm thickness between frames, scales bar: 2

µm. Bottom micrographs: reconstituted 3D volume of the strain, from the Z stack displayed above and using the ImageJ inbuilt 3D project function. See also Supplementary Video 3.

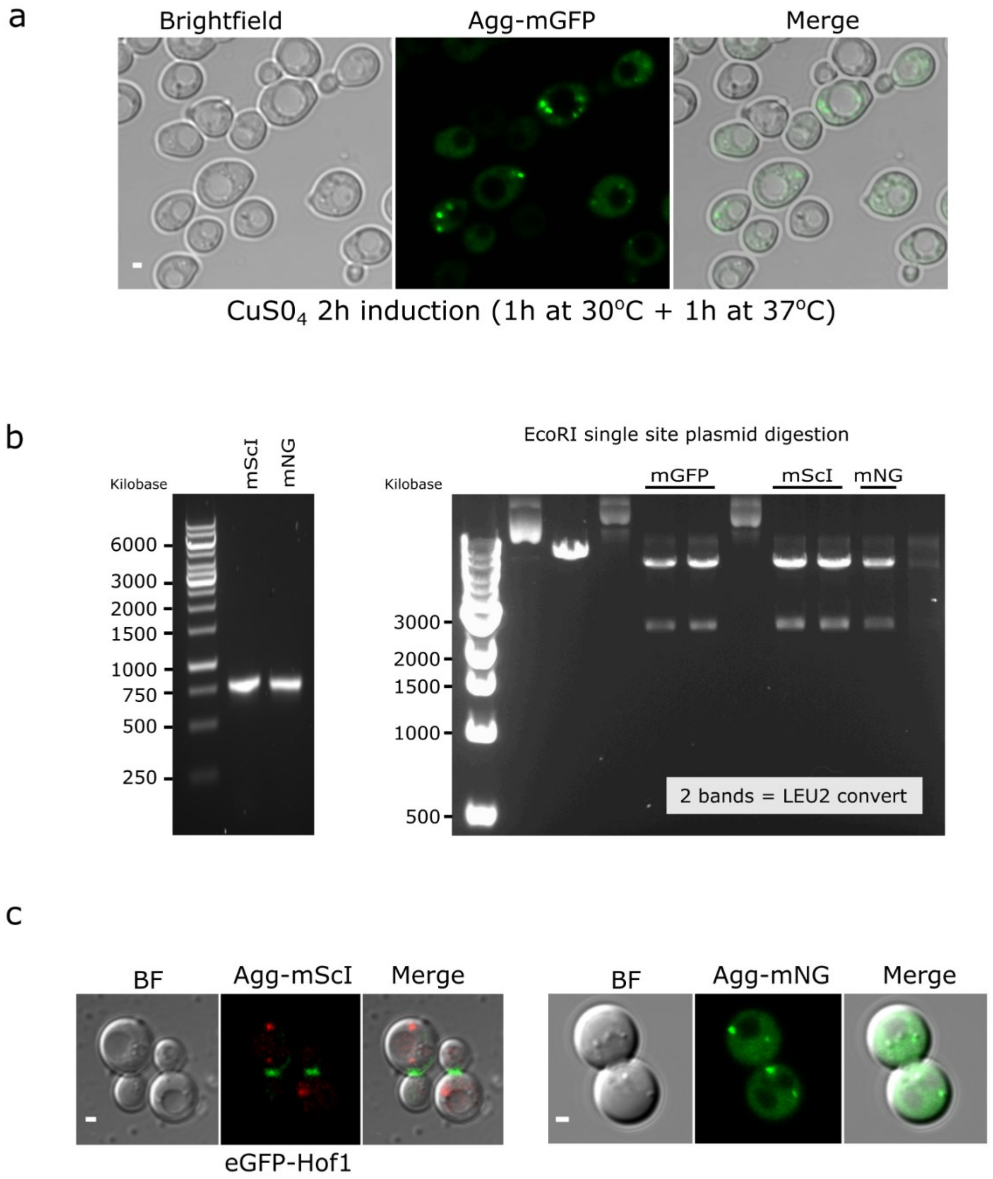

Supplementary Figure 4: ΔssCPY* aggregate, extension of the strain library, LEU convert and dual colour strain.

A) Micrographs representing selected conditions to induce visible protein aggregates under confocal microscopy imaging. B) Electrophoresis gels for the construction of fluorescently tagged ΔssCPY* aggregate with either mScarlet-I or mNeonGreen. On the Left: colony PCR verifying fluorophore exchanged after Gibson assembly, mGFP tag sequence being replaced by either mScarlet-I or mNeonGreen (mNG). On the right: Single digest plasmid with EcoRI to verify plasmid Leu conversion, for mGFP, mScarlet-I and mNeonGreen. Plasmid holding the URA selection only cut once and displaying one band. Plasmid holding the LEU selection will instead display two bands. C) Micrographs mNeonGreen and mScarlet-I version of the ΔssCPY* aggregate reporter. Left: Agg-mScarlet-I expressed in sfGFP-HOF1 strain. Right: Agg-mNeonGreen expressed in BY4742 WT strain.

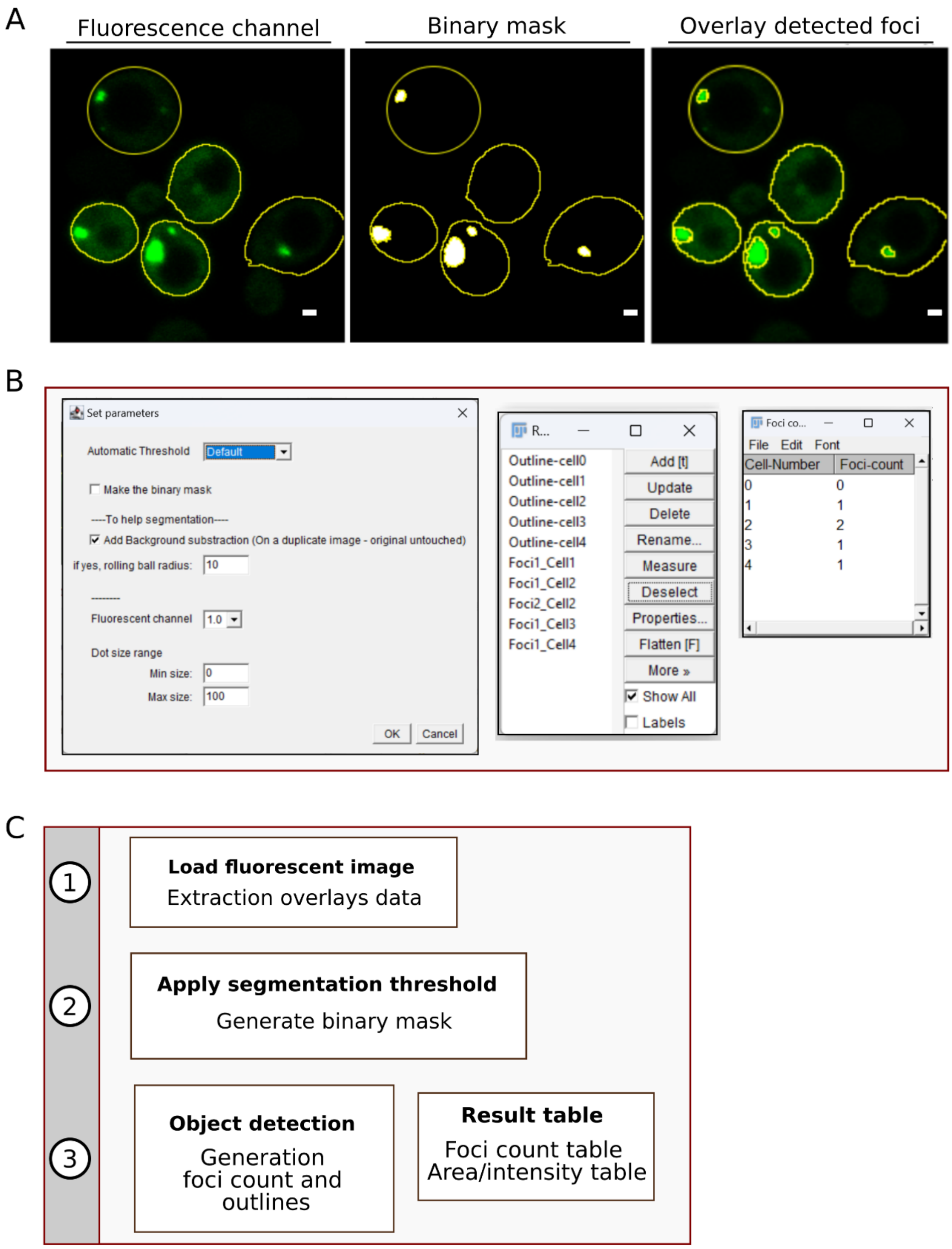

Supplementary Figure 5: ImageJ macro enables automated, objectivity foci quantification for live cell image data. A) Visual generated segmentation via ImageJ object detection function on the generated binary mask. B) Macro user interface and output sport count table. C) Schematic for the foci detection macro workflow.

	30°C	37°C	42°C
% aggregates positive cells	18.98173	47.39776	58.7738
Standard deviation of %	5.871798	4.943365	16.57905
T-test p value (30 vs 37˚C)	7.59×10^{-5}		
T-test p value (37 vs 42˚C)		0.261	
T-test p value (30 vs 42˚C)	5.0×10^{-3}		

Supplementary Table 1: Heat shock and aggregates positive cells

Statistical properties including the p-value taken from Student's *t*-test corresponding to bar plot in Figure3.B

	30°C	37°C	42°C
mean foci count/100cells	26.502	62.644	131.025
Standard deviation of %	9.705	5.035	18.457
T-test p value (30 vs 3˚C)	0.000167		
T-test p value (37 vs 42˚C)		0.00021	
T-test p value (30 vs 42˚C)	2.74×10^{-5}		

Supplementary Table 2: Heat shock and aggregates counts

Statistical properties including the *t*-test p value corresponding to bar plot in Figure 3.C

	Mother cells	Daughter cells
Foci mean area	0.987	0.393
Foci median area	0.822	0.284
Standard deviation foci area	0.744	0.291
Foci mean intensity	52375.69	37138.98
Foci median intensity	51452.68	36550.59
Standard deviation foci intensity	8512.51	9785.60
T-test p value (foci area)	8.60×10^{-6}	
T-test p value (Foci intensity)	3×10^{-14}	

Supplementary Table 3: Comparison aggregates area and intensity properties between mother cells and daughter cells.

Statistical properties including the *t*-test p value corresponding to Jitter plots in Figure 5.B

	vacuole	Nucleus
average correlation percentage (%)	43.94737	68.11404
Standard deviation	36.34713	32.67815
T-test p value	0.0057	

Supplementary Table 4: Correlation aggregates and key subcellular compartments

Statistical properties corresponding to Figure 5.D.
